# Chilaiditi Syndrome Presenting as Partial Colonic Obstruction

**DOI:** 10.7759/cureus.22975

**Published:** 2022-03-08

**Authors:** Eric J Basile, Ammar Ahmed, Eraad Rahman, Omar Rafa, Elisabeth L Frankini, Anthony Modica

**Affiliations:** 1 Internal Medicine, Touro College of Osteopathic Medicine, New York City, USA; 2 Medicine, Global Health, McMaster University, Hamilton, CAN; 3 Medicine, New York Institute of Technology College of Osteopathic Medicine, Old Westbury, USA; 4 Orthopedic Surgery, Touro College of Osteopathic Medicine, New York City, USA

**Keywords:** colonic interposition, bowel obstruction, chilaiditi syndrome, chilaiditi sign, adult gastroenterology

## Abstract

Chilaiditi sign is a rare incidental radiographic finding where bowel is interposed between the diaphragm and the liver, often seen as air under the right hemidiaphragm. A majority of patients with Chilaiditi sign are asymptomatic and remain so throughout their lifetime. Chilaiditi sign is recategorized as Chilaiditi syndrome if it becomes symptomatic and is a very rare etiology of bowel obstruction. As bowel obstruction confers a huge financial burden to the health care system, studies of even the rarer etiologies are of significant value. Particularly in the case of Chilaiditi syndrome, the free air under the right hemidiaphragm can lead physicians to prematurely conclude pneumoperitoneum, which would require an emergent surgical evaluation. It is through the incorporation of a broad differential and clinical presentation that physicians can decrease the inappropriate allocation of hospital resources and unnecessary surgical procedures; additionally, keeping Chilaiditi syndrome on the differential may prevent unnecessary surgical intervention, cost to the patient, and downstream complications. Bowel obstruction secondary to Chilaiditi syndrome is most commonly treated with conservative management including intravenous fluids, bowel rest, decompression, and laxatives. If the symptoms worsen and progress to full bowel obstruction, surgical intervention has shown great efficacy. We report a case of a 69-year-old male who presented to the emergency department for progressively worsening abdominal pain, nausea, and vomiting incidentally found to have colonic interposition with mild colonic dilatation on computed tomography (CT) imaging. The patient was diagnosed with bowel obstruction secondary to Chilaiditi syndrome and treated non-surgically with rapid recovery.

## Introduction

There are an estimated 300,000 hospitalizations per year secondary to intestinal obstruction in the United States, which accounts for approximately 15% of all emergency room admissions. Annually, the financial burden of intestinal obstruction on the healthcare sector is approximately three billion dollars [1]. Large bowel obstruction (LBO) accounts for 10-15% of all intestinal obstructions, with the most common causes being colon carcinoma, diverticular disease, and volvulus [2].

Signs of bowel obstruction include abdominal distention, nausea, vomiting, diarrhea, inability to pass flatus, constipation, loss of appetite, and abdominal pain. Differentiation of small bowel obstruction (SBO) and LBO via clinical presentation alone may be difficult due to overlapping signs, but quality, timing, and presentation can aid in distinguishing between the two; the abdominal pain in LBO is typically continuous and more diffuse, whereas pain secondary to SBO is more colicky and focal in nature. The vomiting that accompanies SBO tends to be more voluminous, bilious, and more frequent than that of LBO. Additionally, the abdominal distention is more marked in LBO than in SBO [2].

The workup for a patient who presents with signs or symptoms of intestinal obstruction includes determining if the patient is clinically stable and performing imaging to see if findings are consistent with obstruction; instability or any indication of vascular compromise, perforation, or closed-loop obstruction are indications for emergent surgical evaluation or operative exploration. A hallmark finding for bowel viscus perforation is pneumoperitoneum - free air under the diaphragm on chest x-ray kidney, ureter, and bladder x-ray (KUB). This, however, should not be the sole determining factor for the determination of need for surgical intervention as there are other causes for air under the diaphragm. One such example is Chilaiditi sign - a benign radiological finding with an incidence of 0.025-0.28%, which describes the interposition of a part of the bowel between the diaphragm and the liver, a finding that can be misinterpreted as pneumoperitoneum [3,4]. The criteria to make the diagnosis of Chilaiditi sign includes the following: a right hemidiaphragm that is elevated above the liver by the intestine, the bowel must be distended by air to illustrate pseudopneumoperitoneum, and the superior margin of the liver must be depressed below the level of the hemidiaphragm [5]. Chilaiditi syndrome refers to the medical condition in which a Chilaiditi sign is accompanied by clinical symptoms. The most common symptoms are that of LBO, which could be accompanied by respiratory distress and anginal chest pain.

The exact cause of Chilaiditi syndrome is unknown, but risk factors include abnormal elongation of the colon, laxity of ligaments in the colon and liver. It has been noted to occur in individuals with chronic lung disease, cirrhosis, and ascites at a greater frequency. Additionally, Chilaiditi syndrome is associated with irritable bowel diseases and Ogilvie syndrome (pseudo-obstruction) [6]. It is typically treated based on the stability of the patient and extent of intestinal obstruction; if the patient is unstable or progresses to full intestinal obstruction, surgical evaluation should be performed immediately. In pediatric patients, minimally invasive colopexy has been used in cases where Chilaiditi syndrome failed to resolve with conservative therapy [7]. Complications of Chilaiditi syndrome include cecal perforation, subdiaphragmatic appendicitis, and volvulus of the cecum, splenic flexure, or transverse colon [4]. We report a case of Chilaiditi syndrome in a 69-year-old male presenting with signs and symptoms consistent with partial LBO.

## Case presentation

A 69-year-old male with a past medical history remarkable for diverticular disease and gastroesophageal reflux disease presented to the emergency department for progressively worsening abdominal pain. The pain was colicky in nature and diffuse. The patient endorsed nausea and a two-day history of constipation. He stated that he was unsure whether or not he passed flatus since the pain started. Upon interview, the patient expressed that he has had similar episodes in the past to a lesser extent. He has no significant past surgical history. He is a non-smoker and drinks two alcoholic beverages per day. The patient had a temperature of 99.4˚F, a heart rate of 88 beats per minute, a blood pressure of 115/84 mmHg, and was tachypneic at 22 breaths per minute.

**Figure 1:**
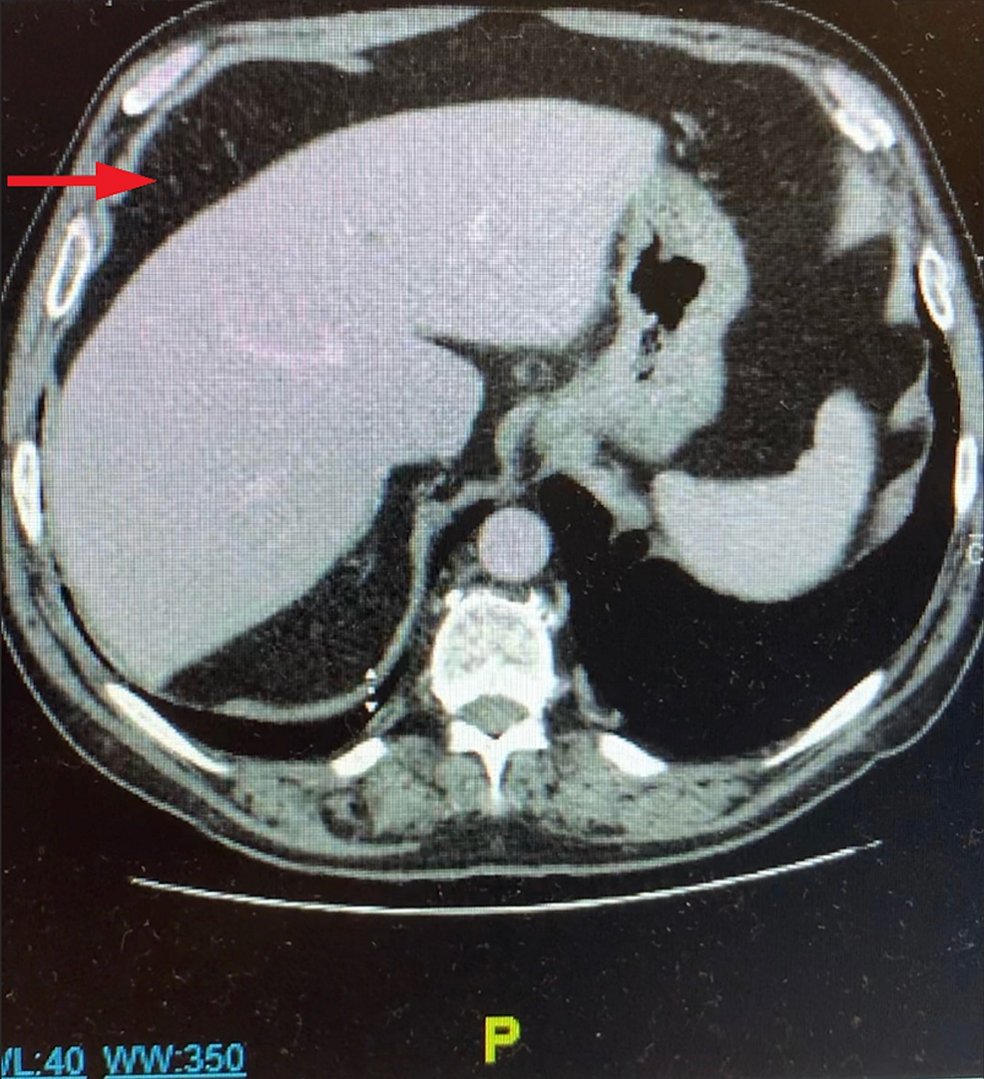
Transverse computed tomography scan without contrast showing colonic interposition between the liver and diaphragm (red arrow).

On physical examination, hyperactive bowel sounds, moderate abdominal distention, and diffuse abdominal tenderness were appreciated. The abdominal tenderness was worst in the right upper quadrant and Murphy’s sign was negative. The pain and nausea worsened when lying supine compared to seated or standing. A right-sided direct inguinal hernia was appreciated, reducible, non-tender, and non-indurated. A complete metabolic panel showed hyponatremia with a serum sodium concentration of 132 mEq/L and a slightly elevated aspartate aminotransferase (AST) of 62 u/L with the rest of the studies within normal limits. Lipase and a complete blood count were also within normal limits. An electrocardiogram showed normal sinus rhythm. A chest x-ray was performed, which was of poor quality due to patient movement, and demonstrated a potential small quantity of air under the right hemidiaphragm. CT scan of the abdomen and pelvis without contrast demonstrated two hypodense lesions in the liver suspicious for cysts, a small hiatal hernia, submucosal fat deposition in the cecum, ascending colon, and transverse colon that favors chronic inflammatory bowel disease, and colonic interposition with mild proximal dilation (Figure 1). The CT scan also demonstrated a right-sided inguinal hernia containing mesenteric fat, unchanged from previous imaging conducted two years prior.

The patient was admitted overnight under observation and made nil per oral (NPO). He was afebrile throughout the entire admission and treated conservatively with intravenous fluids but refused nasogastric tube decompression. The patient was unable to have a bowel movement, but he was able to produce flatus. The patient agreed to a trial of magnesium citrate which yielded a large bowel movement, which significantly reduced the distention and resolved his other symptoms. The patient was able to tolerate a meal and was later discharged to home.

## Discussion

After initial assessment of hemodynamic stability, patients presenting with symptoms consistent with intestinal obstruction are initially worked up through a hierarchy of imaging modalities to assess the cause and need for surgery. Initial plain radiographs may show air below the dome of the right diaphragm. Surgeons unfamiliar with Chilaiditi sign may immediately interpret this as pneumoperitoneum and undergo further surgical exploration without further exploring other differentials, exposing patients to a myriad of potential unnecessary complications when conservative measures may suffice. Given the risks of surgery, clinicians and surgeons should keep a broad differential for air under the diaphragm including subphrenic abscess, intestinal pneumatosis, pneumoperitoneum, and Chilaiditi syndrome among others, especially when the clinical picture does not align with these other working diagnoses.

The main goal when this radiographic finding is noted should be to assess whether the subdiaphragmatic air is free or intraluminal. For instance, if air has accumulated in the large intestine, haustral folds should be visualized and air should be unaffected by positional changes. However, if air has instead accumulated in the peritoneal cavity, a second radiograph with positional changes should reveal air movement. If there is doubt, confirmation can be obtained by CT, which was what occurred in the case of this patient.

There are many potential implications of failing to account for Chilaiditi sign. Misdiagnosis as bowel perforation may expose patients to potential surgical complications. On the one hand, in cirrhotic patients, a failure to recognize Chilaiditi syndrome may lead patients who are undergoing percutaneous transhepatic procedures or liver biopsies to potentially puncture the intestine. There is also increasing evidence that pursuing colonoscopy in these patients can lead to perforation caused by the increased risk of air entrapment in angulated, interposed bowel. Thus, failing to account for colonic interposition may unnecessarily result in a wide array of complications.

## Conclusions

Intestinal obstruction places a substantial financial strain on the healthcare system. It is important both in terms of appropriate allocation of resources and patient outcomes to consider a broad list of differential diagnoses that include more uncommon etiologies. Chilaiditi syndrome is of particular importance as it can often be confused with pneumoperitoneum, potentially leading to an unnecessary surgical evaluation and intervention that may precipitate other sequelae. It is of equal importance to understand how Chilaiditi syndrome may present clinically and predispose to bowel obstruction, which may aid in the prevention of hospitalizations. Further, knowledge of associated conditions and complications for this condition may provide more insight into who is at risk and guide therapeutic options. Further investigation into the etiology, risk factors, clinical presentation, and complications is therefore warranted. A greater body of research literature on this condition will undoubtedly lead to an improved ability to accurately diagnose and manage it while also promoting an optimized use of hospital resources.
